# Maize Flavonoid Biosynthesis, Regulation, and Human Health Relevance: A Review

**DOI:** 10.3390/molecules27165166

**Published:** 2022-08-13

**Authors:** Héctor A. Peniche-Pavía, Tereso J. Guzmán, Jesús M. Magaña-Cerino, Carmen M. Gurrola-Díaz, Axel Tiessen

**Affiliations:** 1 Departamento de Bioquímica y Biotecnología, Centro de Investigación y de Estudios Avanzados del Instituto Politécnico Nacional Unidad Irapuato, Libramiento Norte Km. 9.6, Irapuato 36824, Guanajuato, Mexico; 2 Department of Pharmacology, Institute of Pharmaceutical and Medicinal Chemistry, University of Münster, Corrensstraße 48, 48149 Münster, Germany; 3 División Académica de Ciencias de la Salud, Centro de Investigación y Posgrado, Universidad Juárez Autónoma de Tabasco, Av. Gregorio Méndez Magaña 2838-A, Col. Tamulté de las Barrancas, Villahermosa 86150, Tabasco, Mexico; 4 Departamento de Biología Molecular y Genómica, Centro Universitario de Ciencias de la Salud, Instituto de Investigación en Enfermedades Crónico Degenerativas, Instituto Transdisciplinar de Investigación e Innovación en Salud, Universidad de Guadalajara, C. Sierra Mojada 950. Col. Independencia, Guadalajara 44340, Jalisco, Mexico

**Keywords:** *Zea mays* L., anthocyanins, biosynthesis, regulation, health benefits, pigmented maize

## Abstract

Maize is one of the most important crops for human and animal consumption and contains a chemical arsenal essential for survival: flavonoids. Moreover, flavonoids are well known for their beneficial effects on human health. In this review, we decided to organize the information about maize flavonoids into three sections. In the first section, we include updated information about the enzymatic pathway of maize flavonoids. We describe a total of twenty-one genes for the flavonoid pathway of maize. The first three genes participate in the general phenylpropanoid pathway. Four genes are common biosynthetic early genes for flavonoids, and fourteen are specific genes for the flavonoid subgroups, the anthocyanins, and flavone C-glycosides. The second section explains the tissue accumulation and regulation of flavonoids by environmental factors affecting the expression of the MYB-bHLH-WD40 (MBW) transcriptional complex. The study of transcription factors of the MBW complex is fundamental for understanding how the flavonoid profiles generate a palette of colors in the plant tissues. Finally, we also include an update of the biological activities of C3G, the major maize anthocyanin, including anticancer, antidiabetic, and antioxidant effects, among others. This review intends to disclose and integrate the existing knowledge regarding maize flavonoid pigmentation and its relevance in the human health sector.

## 1. Introduction

The comprehension of the maize flavonoid pathways is necessary not only for plant breeders that want to develop new pigmented maize varieties with better nutraceutical properties but also for any health and food scientists working with phenolic compounds. The diversity in the palette of color in maize seeds correlates with differences in the pigment, including carotenoids and flavonoids. We will deepen into these aspects to explain the impressive correlation between plant color, plant survival, and human health.

**Figure 1:**
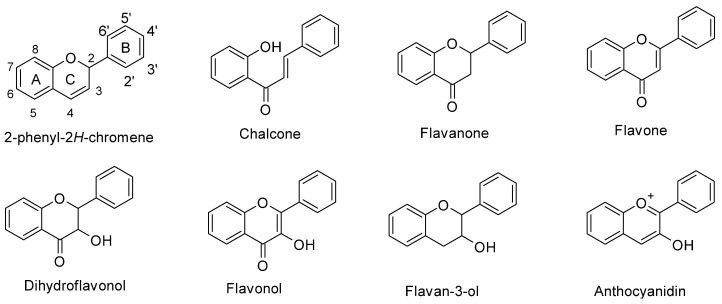
Chemical structure of flavonoid subgroups and the basic C6-C3-C6 skeleton (2-phenyl-2*H*-chromene). A, B, and C refer to a specific ring of the flavonoid skeleton.

In maize (*Zea mays* L.), flavonoids act as deterrents against herbivores, regulate pollen development, and also have defensive roles against UV-B radiation [1,2,3]. Flavonoids are a large family of phenolic compounds that share a biosynthetic pathway and, therefore, a common chemical arrangement. The basic structure consists of a C15 skeleton arranged in a C6-C3-C6 where one of the C6 corresponds to a phenyl that is bound to a benzopyran (C6-C3) denominated chromene according to the IUPAC nomenclature (Figure 1). Flavonoids originate from the mevalonate and phenylpropanoid pathways converging in C6-C3-C6 compounds. Some flavonoid molecules differentiate themselves by the chemical changes in the pyran ring, also known as the flavonoid’s C ring [4,5]. For example, anthocyanidins have a modified benzopyran structure with a double bond between the oxygen atom and C2, forming the flavylium cation. Meanwhile, flavones have a double bond between carbons 2 and 3 and a carbonyl group at the C4 position.

In this review, we organized the current knowledge about maize flavonoids into three sections: the structural genes of the maize flavonoid pathway; the regulatory factors of the pathway; and the health effects of cyanidin-3-*O*-glucoside, one of the most abundant flavonoids in the maize. First, we describe the biosynthesis pathway, addressing the genes involved and their function, including enzymes, transport proteins, and transcription factors. The flavonoid pathway consists of two gene groups: early and late genes [6]. The former group engages in the biosynthesis of common flavonoid intermediates (i.e., chalcones and flavanones). The latter is related to genes implicated in the biosynthesis of specific flavonoid subgroups such as anthocyanins and C-glycosylated flavones. Later, in the regulation section, it is described how the MYB-bHLH-WD40 (MBW) complex regulates the network of biosynthetic genes.

From a historical point of view, the scientific community invested much effort into deciphering the flavonoids’ role as food pigments [7,8]. Nevertheless, reports of new properties convert them into an interesting research subject that contributes to a healthy balance in many organisms; an example is their beneficial role in the intestinal microbiota [9,10]. Furthermore, the protective effect of the consumption of anthocyanins in the development of cardiovascular diseases and other chronic pathologies has been observed [11,12]. Therefore, in the third section, we focus on the most abundant and studied compound in pigmented maize, the cyanidin-3-*O*-glucoside which exerts different biological activities in in vitro and in vivo studies [13,14,15].

## 2. Structural Protein Genes of the Maize Flavonoid Pathway

### 2.1. Phenylpropanoid Pathway

The first enzymatic steps in the flavonoid pathway are from three genes of the phenylpropanoid pathway (Table 1). These three enzymes direct the transformation of phenylalanine to coumaroyl-CoA. Those genes are *ZmPAL* (phenylalanine ammonium lyase, multiples genes, EC 4.3.1.24) [16], *ZmC4H* (cinnamic acid 4-hydroxylase, Zm00001d009858, EC 1.14.14.91) [16,17], and *Zm4CL* (4-coumarate CoA ligase, *bm5*, EC 6.2.1.12) [18]. The three genes share a similar expression profile of downstream genes in the flavonoid pathway in anthocyanin-pigmented tissues [19,20]. Recent analyses have demonstrated multiple gene families in flavonoid biosynthesis with a tissue-specific expression. In addition, some genes such as *Zm4CL* codify various isoforms, each of which has specific functions [21]. The research on these genes focuses on their roles in lignin biosynthesis [18]. For example, under sugarcane mosaic virus (SCMV) infection, *ZmPAL* and *ZmC4H* genes are upregulated, generating the substrate for lignin production [16]. Meanwhile, studies on a brown *midrib5* maize line demonstrated that a *Zm4CL* mutant was responsible for a defective lignin biosynthesis [22]. There is a correlation between anthocyanins and lignin where the fungi *Ustilago maydis* activates the anthocyanin but reduces the lignin biosynthesis, thus facilitating its invasion into the maize seed [23]. 

### 2.2. Early Biosynthetic Genes of Flavonoids

#### 2.2.1. Chalcone Synthase (*ZmCHS*, *c2*, EC 2.3.1.74)

**Figure 2:**
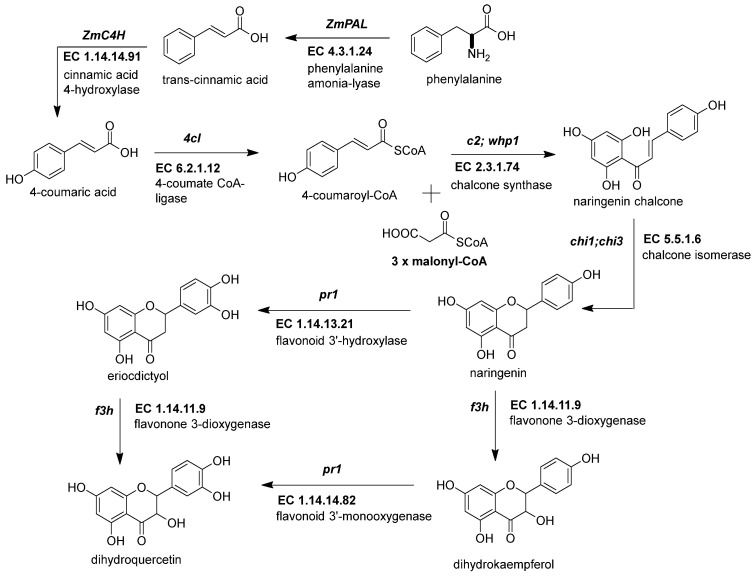
Early genes in the flavonoid pathway. The flavonoid pathway begins with the transformation of phenylalanine to coumaroyl-CoA. The last steps end with the intravacuolar accumulation of acylated anthocyanins. The genes responsible for supplying the coumaroyl-CoA into the flavonoid pathway are phenylalanine ammonium lyase (*ZmPAL*, EC 4.3.1.24), cinnamic acid 4-hydroxylase (*ZmC4H*, EC 1.14.14.91), and 4-coumarate CoA ligase (*Zm4CL*, *bm5*, EC 6.2.1.12). The flavonoid genes are divided into early biosynthetic genes (*EBGs*) and late biosynthetic genes (*LBGs*). EBGs comprise four genes: chalcone synthase (*ZmCHS*, *c2*, EC 2.3.1.74), chalcone isomerase (*ZmCHI*, *chi1*, EC 5.5.1.6), flavonoid 3-dioxygenase (*ZmF3H*, *fht1*, EC 1.14.11.9), and flavonoid 3′-monooxygenase (*ZmF3*′*H*, *pr1*, EC 1.14.14.82). References: [30,32].

The first crucial step in flavonoid biosynthesis (Figure 2) is the production of the naringenin chalcone (C6-C3-C6) from the condensation of three molecules of malonyl-CoA (3 × C2) using a 4-coumaroyl-CoA (C6-C3) as substrate [31]. This gene is also known as polyketide synthase (PKS) type III. The chalcone synthase (CHS) works similarly to other PKS enzymes from the mevalonate/acetate pathway [4]. The reaction extends the aliphatic chain from the coumaroyl-CoA three times using two carbon units from a malonyl-CoA. Then, an intramolecular Claisen condensation occurs to form the second aromatic ring. 

Genome-wide analysis revealed up to 15 *ZmCHS* genes in the maize genome (Han et al., 2016). However, the members more consistently studied are the duplicated *c2* (*ZmCHS01*) and *whip1* (*ZmCHS02*) genes (Table 1) [24]. Multiple tissues, including tassels, ear husks, and aleurone layer of endosperm at different developmental stages, express the genes *c2* and *whip1* [33,34]. Indeed, functional alleles for genes *c2* and *whip1* are vital to increasing the biosynthesis of any flavonoids downstream, such as apigenin and tricin, essential for lignin formation, and C-glycosyl flavones [25,35]. Meanwhile, members of the chalcone synthase family, such as *ZmCHS013* and *ZmCHS014*, compared to *ZmCHS01*, had a lower expression in most tissues and different responses under the stimuli of salicylic acid [36].

#### 2.2.2. Chalcone Isomerase (*ZmCHI*, *chi1*, EC 5.5.1.6)

This enzyme catalyzes an intramolecular Michael-type addition from the chalcone 2-*O* to its α,β-unsaturated carbonyl (Figure 2). The final product is the typical phenyl-chromanone or flavanone structure [4]. The first gene sequenced from this family in maize was *ZmCHI* (Table 1) [26]. Interestingly, mutants have not been reported in maize for this gene, due to the multiple homologous sequences found for *ZmCHI* in the maize genome. An experiment designed to find QTLs for resistance to *Fusarium* corn fungi detected *ZmCHI3* as a second member of the family [37]. Indeed, a transformed maize callus with a copy of *ZmCHI3* from a resistant inbred was less susceptible to maize plagues. 

#### 2.2.3. Flavonoid 3-Dioxygenase (*ZmF3H*, *fht1*, EC 1.14.11.9)

*ZmF3H* is a Fe^2+^ and 2-oxoglutarate-dependent dioxygenase that introduces a hydroxyl group in position 3 of the chalcone structure, generating a dihydroflavonol [27]. There is just one gene copy known in the maize genome. In a previous report, *ZmF3H* was found to be the only gene in the flavonoid pathway in which mRNA expression levels correlate with the synthesis of flavanols in anthers [38]. Moreover, its expression increases in pigmented kernels compared to white seeds [19].

#### 2.2.4. Flavonoid 3′-Monooxygenase (*ZmF3*′*H*, *pr1*, EC 1.14.14.82)

This *pr1* or purple aleurone1 gene has been studied in maize because its alleles are responsible for changes in color pigmentation caused by a difference in anthocyanin profile [39,40]. *ZmF3*′*H* is monooxygenase hydroxylate in the 3′ position from the phenyl ring B (Table 1). When the gene is functional, its enzyme can produce the blue/violet-colored anthocyanidins (cyanidin and peonidin). If not, it generates a red/orange mono-hydroxylated pelargonidin [28]. Red kernels are homozygous for the recessive alleles *pr1* that do not produce functional enzymes, resulting in the pelargonidin-base anthocyanins predominating over the anthocyanin profile. The dominant *Pr1* alleles have a gene dose effect in the purple kernel pigmentation, which means that each *Pr1* allele in diploid (vegetative) or triploid (endosperm) tissues increases the cyanidin-base anthocyanins (Figure 2) in the pigmented tissue [41]. 

Moreover, *ZmF3*′*H* has a role in the biosynthesis of 3-deoxyflavonoids and phlobaphene; as occurs with the anthocyanins, the precursor transforms into a di-hydroxylated phenyl ring B compound [42]. A *Pr1* allele is essential for the resistance against biotic stress depending on C-glucosyl flavone (maysin) accumulation in salmon-colored silks [43]. 

### 2.3. Late Biosynthetic Genes of Maize Anthocyanins

#### 2.3.1. Dihydroflavonol 4-Reductase (*ZmDFR*, *a1*, EC 1.1.1.219)

**Figure 3:**
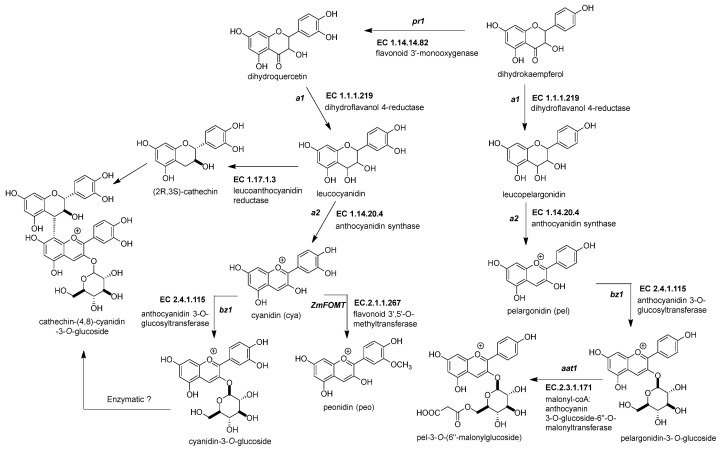
Biosynthetic genes for maize anthocyanin pathway. After the formation of the dihydroflavonol, five enzymatic steps catalyze its biotransformation into acylated maize anthocyanins. Those genes are the following: dihydroflavonol 4-reductase (*ZmDFR*, *a1*, EC 1.1.1.219), anthocyanidin synthase (*ZmANS*, *a2*, EC 1.14.20.4), anthocyanidin 3-*O*-glucosyltransferase (*ZmAGT*, *bz1*, EC 2.4.1.115), malonyl-CoA: anthocyanin 3-*O*-glucoside-6′′-*O*-malonyltransferase (*Zm3MAT*, *aat1*, EC 2.3.1.171), and flavonoid 3′,5′-*O*-methyltransferase (*ZmAOMT*, EC 2.1.1.267). The glutathione S-transferase (*ZmGST*, *bz2*, EC 2.5.1.18) and multidrug resistance protein (*ZmABCC3* and *ZmABCC4*, MRP3 and MRP 4, EC 7.6.2.2) are required to deliver them inside the vacuole. References: [30,32].

This enzyme converts the dihydroflavonol (or flavanonol) to a flavan-3,4 diol by reducing the 4-carbonyl (Figure 3 and Table 2) [44]. There is a hypothesis that this enzyme has a role in phlobaphene biosynthesis by transforming the 4-carbonyl into flavanones to produce 4-flavan-4-ol [45]. The gene locus of *ZmDFR*, a1, has been deeply studied for two reasons. The first is its linkage to the *sh2* gene, responsible for the shrunken seed phenotype, that made possible the studies on transposable elements and meiotic recombination hotspots in the a1-sh2 interval [46,47]. The second reason is that the gene product is a vital enzyme in the flavonoid pathway, favoring which flavonoid subgroup could be biosynthesized [48]. If there is a functional allele, it can produce anthocyanidins (Figure 3) and phlobaphenes (see Section 2.4.1). However, two copies of a non-functional allele would redirect it to flavanol and flavone biosynthesis [49]. 

The role of *ZmDFR* in the diversification of flavonoids is further exemplified by its interaction with multiple transcription factors [19,20]. The identity and function of those transcription factors are discussed in Section 3.1. *ZmDFR1* has a gene duplication in the maize genome, known as *a4*. Nevertheless, it is not clear if there is the active protein in the tissue from the genomic sequences alone [44]. Both genes have a higher expression in pigmented kernels than in anthocyanin-less seeds [19]. 

#### 2.3.2. Anthocyanidin Synthase (*ZmANS*, *a2*, EC 1.14.20.4)

The dioxygenase *ZmANS* oxidizes at the C-3 position of a flavan-3,4 diol, generating a flavan-3,3,4 triol (Figure 3) [27]. After oxidation, two water molecules are removed, producing an anthocyanidin molecule [57]. Moreover, the *ZmANS gene* expression is upregulated in pigmented kernels compared to white seeds through elements that conserve the promoter region for the MBW complex [19,50]. The *a2* is the unique copy known in the maize genome. 

#### 2.3.3. Anthocyanidin 3-*O*-Glucosyltransferase (*ZmAGT*, *bz1*, EC 2.4.1.115)

This enzyme is also known as UDP-flavonoid glucosyltransferase (*ZmUFGT*). It catalyzes the transference of glucose to the C-3 position of anthocyanidins (Figure 3) [58,59]. This locus is named bronze1 since *bz1* alleles cannot produce a functional gene product and are responsible for the bronze-colored aleurone [51,60]. Glycosylated anthocyanidins (anthocyanins) accumulate in a vacuole only when the *ZmAGT* is functional. If not, the anthocyanidins are prone to oxidation, turning into brown pigments in the cell wall [61]. The expression occurs in all anthocyanin pigmented tissue because it contains conserved elements in its promoter, as other genes are upregulated simultaneously by the MYB-bHLH-WD40 (MBW) complex [60,62].

The locus *bz1* is located in the intergenic region *bz1-stc1*, known for the varying copies of transposable elements [63,64]. A relevant study included the first discovery of the first DNA transposable element, the Ac/Ds transposon, that resulted in a Nobel Prize being awarded to Dr. McClintock [65]. The Ds activation by marker Ac produces a chromosome rupture of chromosome 9 short arm region, which was recognized phenotypically by the apparition of bronze-colored spots in the kernel [66].

#### 2.3.4. Malonyl-CoA: Anthocyanin 3-*O*-Glucoside-6′′-*O*-Malonyltransferase (*Zm3MAT*, *aat1*, EC 2.3.1.171)

Two types of acyl moieties can modify the glycosidic part of the anthocyanins in the *Plantae* kingdom, aromatic and aliphatic dicarboxylic acids. *Zm3MAT* (Figure 3) was the first anthocyanin acyltransferase (AAT) discovered not only in maize but also in monocots [52,67]. *Zm3MAT* is necessary to produce mono-malonylated anthocyanins, the most common type of anthocyanins in the aleurone layer [68]. *Zm3MAT* was selected as a QTL for the reduced acylation phenotype and then corroborated through a knockout by Mu transposon insertion [52]. Further research showed that *Zm3MAT* exerts a dimalonyl transferase activity and can utilize both acyl moieties malonyl-CoA and succinyl-CoA, but it is more specific for malonyl-CoA [67]. The spectrum of anthocyanin selectivity ranges from the most preferable to the least preferable as follows: cyanidin-3-*O*-glucoside, pelargonidin-3-*O*-glucoside, peonidin-3-*O*-glucoside, and delphinidin-3-*O*-glucoside.

#### 2.3.5. Flavonoid 3′,5′-*O*-Methyltransferase, or Anthocyanin S-Adenosyl-l-Methionine-Dependent *O*-Methyltransferase (*ZmFOMT* or *ZmAOMT*, EC 2.1.1.267)

This enzyme catalyzes the methylation of a hydroxyl group in the -3′ or -5′ position of the 3-hydroxyflavonoid’s phenyl B-ring (Figure 3) [53]. The enzyme uses several flavonoids as substrates, not just anthocyanins. These include aglycone and glycosylated forms of flavonols or anthocyanidins. However, every member has a specific affinity that favors some substrate above others [68]. Unfortunately, in maize, this enzyme has not been characterized yet. However, Chapman and collaborators mentioned two candidate genes, namely *omt1* (Zm00001d052841) and *omt4* (Zm00001d05284), for anthocyanin *O*-methyltransferases related to QTLs for peonidin-base anthocyanins [54]. 

#### 2.3.6. Glutathione-S-Transferase (*ZmGST*, *bz2*, EC 2.5.1.18)

The glutathione S-transferase (GST) family in maize includes more than 40 GST gene sequences [69]. This family of enzymes detoxifies cells affected by xenobiotics, such as herbicides, by conjugating a glutathione (GSH) molecule [70,71,72]. After being labeled with glutathione, these molecules are sent out of the cell by an ATP-dependent glutathione conjugate export pump [73]. However, the *bz2* gene, a GST type III, is supposed to label the anthocyanin to be recognized by a vacuolar glutathione pump, and then the labeled anthocyanin is transported into the vacuolar lumen [55,73]. Until now, there is no evidence that shows that anthocyanins are conjugated with GSH. However, the role of *bz2* in the accumulation of anthocyanins is accepted. Other authors suggested that this enzyme may function as a carrier protein for vacuolar anthocyanin sequestration [74].

When *ZmGST* is not functional, the anthocyanins are not transported to the vacuole interior. Then, the intravacuolar pH and environment contribute to maintaining these molecules without degradation [75]. As described for *bz1*, a maize plant without functional alleles will develop a bronze-colored kernel [61]. *ZmGST* is upregulated in pigmented tissue because it shares conserved binding sites in the promoter region for the MBW complex interaction, a characteristic shared with other upstream genes in the flavonoid pathway [19,76].

#### 2.3.7. Multidrug Resistance Protein (*ZmABCC3* and *-4*, *mrpa3*, EC 7.6.2.2)

*ZmABCC3* is part of a broader ATP-binding cassette (ABC) superfamily protein containing up to 130 open reading frames [72]. In maize, this superfamily of transmembrane proteins anchored to the cell membrane is highly specialized in expelling xenobiotics from the intracellular environment [77]. However, *ZmABCC3* and *ZmABCC4* are present in the tonoplast of vegetative tissues and in the aleurone layer, respectively [56]. 

This protein follows a similar expression profile to other genes related to anthocyanin biosynthesis [19,78]. Recent research in species such as *Vitis vinifera* and *Arabidopsis thaliana* shows that their homologous sequences to *ZmABBC3* are GSH/anthocyanin co-transporters [79,80].

#### 2.3.8. Flavanol-Anthocyanin Condensed Forms

The flavanol-anthocyanin condensed forms are compounds found in maize; however, there is still no description of a known enzyme producing them [81]. Their biosynthesis starts with the generation of the flavan-3-ol unit (Figure 3). The leucoanthocyanidin reductase (E.C. 1.17.1.3) participates in a reduction reaction in the C-3 position of the leucoanthocyanidin [54,82]. This enzyme is yet unidentified in maize. Then, a linkage occurs between the anthocyanin and the flavan-3-ol, but there is no recognized enzyme for this process (Figure 3). However, it is known that a QTL for the flavanol-anthocyanin condensed form was mapped near the *p1* locus [54]. 

In wine, the presence of flavanol-anthocyanin condensed forms is related to aging. However, in maize, there is evidence of natural formation [81]. The production of flavanol-anthocyanin condensed forms consumes monomeric anthocyanin, therefore reducing the total concentration [67].

### 2.4. Biosynthesis of Flavonols, Flavones C-Glycosides, and Phlobaphenes in Maize

#### 2.4.1. Flavonol Synthase (*ZmFLS1*, *fls1*, EC 1.14.20.6)

**Figure 4:**
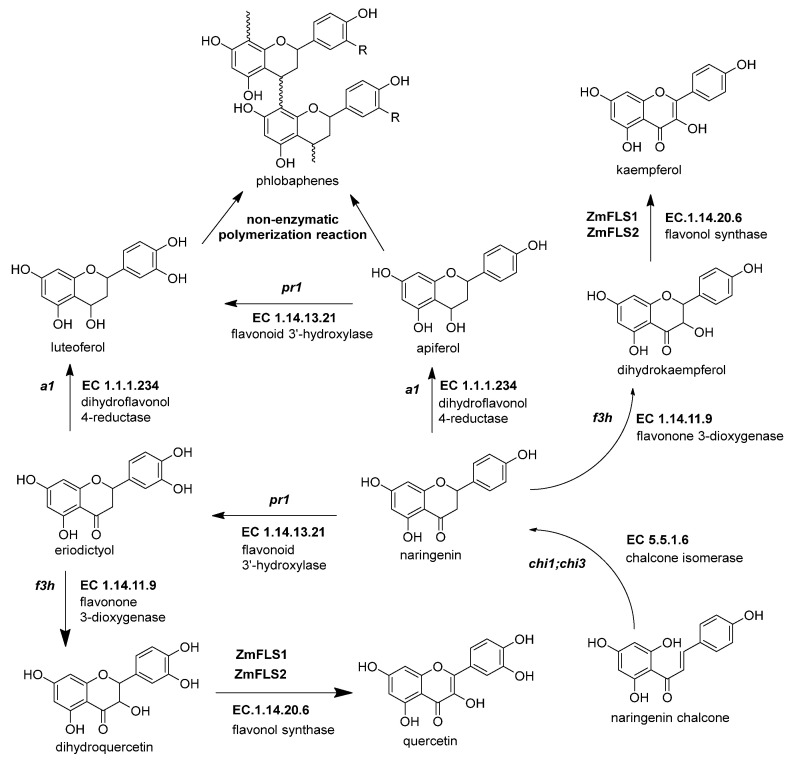
The biosynthetic genes of flavonol and phlobaphenes. The flavanones naringenin and eriodyctiol are the starting substrates for the other flavonoid subgroups. Flavonol synthesis depends on flavanone 3-dioxygenase (*ZmF3H*, *fht1*, EC 1.14.11.9) and flavonol synthase (*ZmFNS1*, *fns1*, EC 1.14.20.5). Phlobaphene synthesis begins with the action of dihydroflavonol 4-reductase (*ZmDFR*, *a1*, EC 1.1.1.219) on flavanones, generating flavan-4-ol molecules that undergo a non-enzymatic polymerization into phlobaphenes. References: [8,30,32].

The flavonols are important in maize due to their effects on male fertility and UV-B protection [83]. Flavonol synthesis depends on flavanone 3-dioxygenase and flavonol synthase, a Fe^2+^/2-oxoglutarate dependent dioxygenase (Figure 4 and Table 3). The transcription factors that regulate the expression of anthocyanins and C-flavone glycosylated biosynthetic genes can also upregulate the expression of *ZmFLS1* [1,27]. In the maize genome are two copies (*ZmFLS1* and *ZmFLS2*) in tandem in the long arm of chromosome 5. The expression of both enzymes was augmented under UV-B light and in high-altitude landraces compared to the inbred lines through an increased *p1* expression [1,3].

#### 2.4.2. Flavone Synthase I (*ZmFNSI1-2*, *fnsi1*, EC 1.14.20.5) and Flavone Synthase II (*ZmFNSII-1*, *fnsii1*, EC 1.14.19.76)

**Figure 5:**
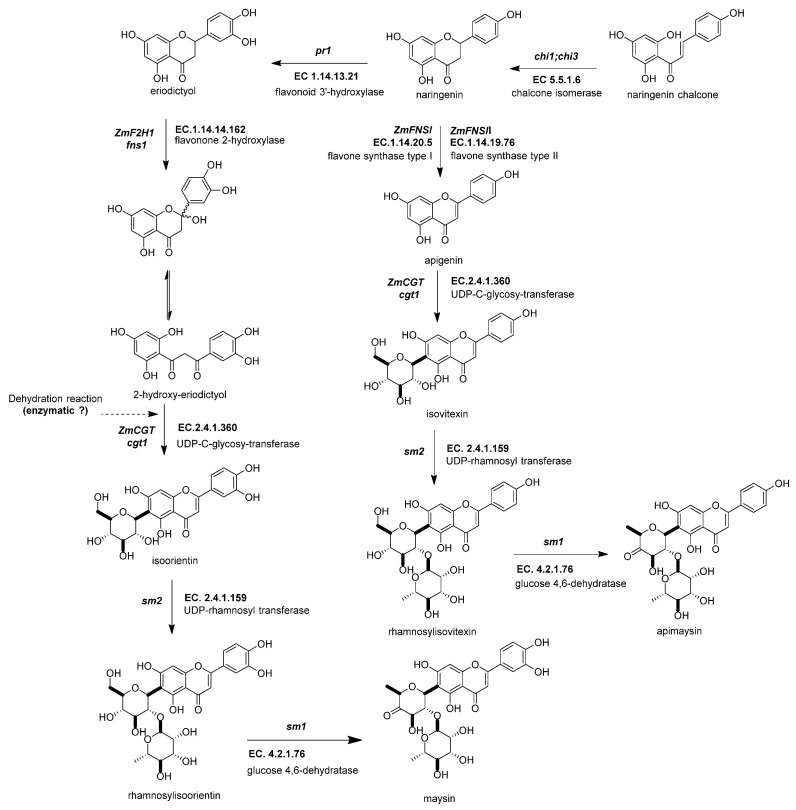
Biosynthetic genes of flavone C-glycosides. The flavanones naringenin and eriodictyol are the initial substrates for the other flavonoid subgroups. There are two possible ways to generate C-glycosyl flavones, indirectly or directly, from any flavanone. The indirect pathway begins through flavanone-2-hydroxylase (*ZmF2H*, *fnsii1*, EC 1.14.14.162) opening the C-ring, producing a 3-oxo-dihydrochalcone. Then, UDP C-glycosyl transferase (*ZmCGT*, *cgt1*, EC 2.4.1.360) generates a glycosidic bond in the A-ring. Then, there is a dehydration reaction (spontaneous or enzymatic) that produces the C6-flavone glycoside. The direct pathway firstly involves flavone synthase I (*ZmFNSII-2*, fnsii2, EC 1.14.20.5) and flavone synthase II (*ZmFNSII-1*, *fnsi2*, EC 1.14.19.76) producing the same reaction by the addition of a double bond between C2 and C3 in the flavanone. Then, a flavone functions as a substrate for the UDP C-glycosyl transferase (*ZmCGT*, *cgt1*, EC 2.4.1.360). The enzymatic action of UDP-rhamnosyl transferase (*ZmCGT*, *sm2*, EC 2.4.1.159) and glucose 4,6 dehydratase (*sm1*, EC 4.2.1.76) produces either apimaysin or maysin. References: [30,43,90].

Maize possesses three enzymes that can synthesize flavones from a flavanone, flavone synthases I and II and flavone 2-hydroxylase (Figure 5) [2,86]. The flavone synthase produces a desaturation in the C2–C3 bond in the flavanone through an oxidation reaction. The oxidative mechanism in *ZmFNSI* is a Fe^2+^/2-oxoglutarate-dependent dioxygenase, like in *ZmFLS1*, whereas that in *ZmFNSII* is CYP450 [2]. In addition, *ZmFNSI1* is upregulated more in tassels than in silks compared to *ZmF2H* [88]. The *p1* transcription factor regulates the expression of *ZmFNSI*. Meanwhile, the anthocyanin MBW complex regulates the expression of *ZmFNSII*. Both types of flavone synthases generate apigenin, which defends the plant against UV-B radiation-induced damage [2].

#### 2.4.3. Flavanone 2-Hydroxylase (*ZmF2H1*, *fns1*, EC 1.14.14.162)

In maize, this is the third known enzyme that can produce the flavone backbone of the C-flavone glycosides in the salmon-colored silks [85]. This enzyme is phylogenetically closer to FNS type II, both being CYP proteins [86,89]. Flavanone-2-hydroxylase adds a hydroxyl group into the flavanone C-2, producing the opening of the C-ring and finally generating the 3-oxo-dihydrochalcone (Figure 5). After this opening, it can be glycosylated in either of the two positions of the A-ring, closing the C-ring, eliminating water (spontaneous or not), and then generating in vitro a mixture of C-6 or C-8 glycosylated flavones [86]. 

#### 2.4.4. UDP-Glucose:2-Hydroxyflavanone C-Glucosyltransferase (*ZmCGT*, *cgt1*, EC 2.4.1.360)

UDP-glucose:2-hydroxyflavanone C-glucosyltransferase generates a glycosidic bond in the A-ring from the C-1 of the glucose to the C-6 in the C-glycosyl flavones (Figure 5) [43]. In vitro and in vivo experimental evidence has demonstrated that the *ZmCGT* enzyme has a bifunctional capacity to form glycosidic bonds with C or O atoms. On the contrary, there is only in vitro evidence for C-8 flavone glycosides [85]. The likely reason for that is the possibility of an enzyme that only selects C-6 glycosylated 2-hydroxyflavanone for dehydration into C-6 glycosyl flavones [91].

#### 2.4.5. UDP-Rhamnosyl Transferase (*sm2*, *UGT91L1*, EC 2.4.1.159)

The UDP-rhamnosyl transferase enzyme forms the glycosidic bond between the glucose C-2 and the rhamnose C-1 (Figure 5) [87]. Functional alleles confer a characteristic salmon color to the silks due to the accumulation of maysin/apimaysin in the silks. This is due to *p1* upregulating *sm2* and is expressed principally in silks [43] but also in non-vegetative tissues such as pollen, tassels, and seeds [88].

#### 2.4.6. Glucose-4,6 Dehydratase (*ZmRHS1*, *sm1*, EC 4.2.1.76)

The biosynthesis of C-flavones glycosides in maize ends with a modification to the glucose structure of the rhamnosylisoorientin (or rhamnosylisovitexin) to produce maysin/apimaysin (Figure 5) [92]. These metabolites give the ear of maize the ability to deter the herbivore *Helicoverpa zea*, commonly known as corn earworm [25,43]. This locus was found to be responsible for producing the last step in the maize flavone pathway and found to be a putative UDP-rhamnose synthase (*ZmRHS1*) [43,87]. The gene has two putative domains; the first domain is a UDP-glucose dehydratase, and the second domain corresponds to UDP glucose 4-keto-6-deoxyglucose epimerase/reductase. The former domain is the exclusive one catalyzing maysin or apimaysin biosynthesis [43]. Its gene expression pattern in the tissues is similar to the *sm1* profile [2].

## 3. Regulatory Factors of the Maize Flavonoid Pathway

We will focus in this section on the genetic regulation of the two flavonoid subgroups in maize with the most relevance to the scientific community: anthocyanins and C-glycosylated flavonoids. Maize has been and continues to be a model organism for studying the genetics of anthocyanin accumulation and its regulation in plants. Therefore, most of the information generated on flavonoid regulation has been focused on one subgroup, and we will also focus primarily on anthocyanins. Flavonoid regulation must be related to the role of the MYB family transcription factors and, in the particular case of anthocyanins, the function of the MBW transcriptional complex [93]. This set of genes constitutes the key to flavonoid regulation in many plant species, not only in maize.

In Section 3.1, we will describe how the MBW complex regulates the expression of the enzymatic machinery of maize flavonoids. Later, in Section 3.2, it will be explained how the environment and biological factors (e.g., changes in the concentration of phytohormones) regulate the expression of the aforementioned transcription factors [94]. Although it is not yet possible to have a complete overview of the regulation of flavonoids, we will present a concise and updated description of this topic.

### 3.1. The MBW Complex

We begin this section by explaining the R2R3-MYB genes involved in anthocyanin biosynthesis (Table 4). A comprehensive genome-wide analysis for this family revealed the involvement of a total of 157 genes (Du et al., 2012), and according to the GRASSIUS server [95], a total of 167 gene sequences. The most comprehensively studied MYB genes in maize are the two pairs of paralogs in the list of genes (Table 4); the first couple is *c1* (*ZmMYB1*) and *pl1* (*ZmMYB2*), and the second couple is *p1* (*ZmMYB3*) and *p2* (*ZmMYB55*) [96,97,98].

The *c1* and *pl1* genes regulate the accumulation of anthocyanins [104,105] by upregulation of LBGs of these molecules [6]. The former is expressed only in the triploid aleurone layer and scutellum [106], and the latter in the diploid vegetative tissue, including the pericarp [107]. These MYB proteins only function in the complex MBW [108]. However, *p1* and *p2* accumulate 3-deoxyflavonoids, flavones, and phlobaphenes, without bHLH and WD40 proteins [42,43,109]. The *p1* expression is performed in pericarp, cob, and tassel glumes, whereas *p2* expression controls the accumulation of anthocyanins of anthers, and both genes produce the salmon-colored silks to repel corn earworm [110,111]. The effect of *p1* on the accumulation of phlobaphenes correlates with an augmented thickness of the pericarp and lower mycotoxin levels [112]. 

The second member of the MBW complex is from the bHLH family. In the GRASSIUS database, there are a total of 175 sequences for maize. However, a recent genome-wide analysis for the bHLH family in maize found up to 208 *ZmbHLH* gene sequences [90]. The first one to be studied was the *r1* gene (*ZmbHLH1*) due to the observed effects of its diverse alleles, with multiple isoforms, on the regulation of anthocyanin pigmentation in anthers, scutellum, and the aleurone layer of endosperm [99,113,114]. The classification of the alleles is according to their role in the pigmentation of the aleurone and plant. These alleles are R-R (pigmentation in both), R-g (only in the aleurone), r-r (only in the plant), and r-g (non-functional allele) [114]. Meanwhile, the *b1* gene (*ZmbHLH2*) modulates pigment accumulation in vegetative tissue, including the pericarp kernel, as in the case of the high-altitude purple maize varieties [115,116]. 

The *in1* gene represents a classical gene that was supposed to express a bHLH protein. However, the genome-wide analysis of Zhang and collaborators did not identify it as a member of the bHLH family [90]. The dominant version of this gene inhibits the accumulation of anthocyanins in the aleurone tissue by downregulating the expression of the LBGs of anthocyanins compared to the wild type [117]. The proposed mechanism explains that it is through a direct competition against R1/B1 proteins that conform the MBW complex [118]. The locus *a3* is known for negatively regulating the expression of *b1* if a dominant A3 allele is present; however, aside from its genomic location, little is known about its mechanism and gene product [119]. 

The third member of the MBW complex is the *pac1* gene that produces a protein with a WD40 repeat (WDR) motif that enables its capacity for DNA interaction [120]. Mutants of this gene are not as common as other members of the ternary complex. Notably, this is the only member of the ternary complex without a known gene duplication [8,118].

### 3.2. Regulation of MBW Complex

**Figure 6:**
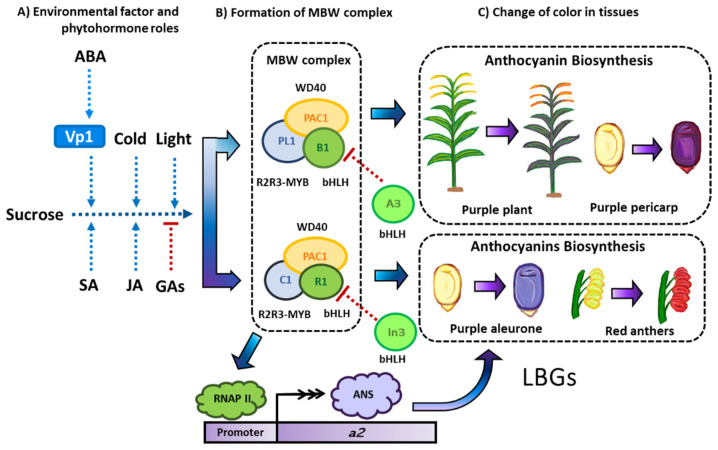
The regulation of the MBW complex and its influence on anthocyanin biosynthesis. (**A**) Environmental factors such as ultraviolet light (UV) and cold temperatures and phytohormones such as abscisic acid (ABA), salicylic acid (SA), and jasmonic acid (JA) augment the expression of the MBW complex. Meanwhile, the gibberellins (GAs) downregulate the transcription of this tripartite complex. In the case of GAs and ABA, their concentrations participate in seed development. In the seed dormancy period, ABA levels increase and the aleurone starts to accumulate anthocyanins. Mutations in the *vp1* gene produce embryos insensitive to ABA, suppressing the anthocyanin biosynthesis in the aleurone and resulting in a viviparous phenotype. (**B**) The complete MBW is necessary to activate the anthocyanin biosynthetic genes. Some gene products such as A3 and In1 compete with the bHLH member of this transcriptional complex, suppressing the anthocyanin accumulation. (**C**) The anthocyanin accumulation modifies the color of the plant’s tissues, turning the vegetative tissues, aleurone, and pericarp into a purple color and turning the anthers into a red color.

Different environmental stimuli may influence the regulatory activities of MBW complexes, such as light, temperature, water/nutrient deficiencies, and other internal stimuli related to changes in hormone levels [94]. Unfortunately, most recent studies on regulation do not focus on *Z. mays* (Figure 6) but focus on model organisms such as *Petunia* and *Arabidopsis* [121]. However, there is relevant information about the effects of the environment on MBW regulation in maize. For example, cold temperatures (10 °C) in seedlings increase not only the expression levels of genes such as *ZmPAL* (8-fold) and *ZmCHS* (50-fold), but also those of anthocyanin biosynthetic and regulatory genes (up to 7- to 10-fold) [122]. Similarly, light stimulates the expression of *r1* (Hopi allele) and *c1* and induces the accumulation of anthocyanins in the aleurone and scutella during seed maturation and germination, respectively [123]. Moreover, anthocyanins have a role in the protection from the photo-inhibition of UV light. Therefore, it is not surprising that the expression of functional alleles for *pl1* and *r1* increases in seedlings under ultraviolet, blue, and white light treatments compared to that of the null alleles [124,125]. 

Both light and temperature induction of anthocyanins require the co-supply of sucrose or other sugars [94]. This sucrose induction of anthocyanins works in conjunction with phytohormones (Figure 6). Both jasmonic acid and abscisic acid (ABA) stimulate the accumulation of anthocyanins in maize tissue. Specifically, ABA enhances the expression of c1 from the MBW complex [126,127]. ABA is also necessary for the maturation of maize seeds. When there is no ABA production in the kernel, like in the *vp1* mutation, the embryo does not become dormant, and there is a suppression of anthocyanin pigmentation in the aleurone [128]. Contrarily, gibberellic acid (GA) exerts an inhibitory effect on anthocyanin concentrations; maize lines modified to suppress GA production have augmented anthocyanin accumulation [129]. Cytokinins enhance the anthocyanin biosynthesis under light conditions but decrease it in the achlorophyllous tissue [124,127]. Whitefly (*Bemisia tabaci* (Genn.)) infestation induced a strong response against drought stress in maize, enhancing the expression of jasmonic acid (JA) and anthocyanin biosynthetic genes in the roots and leaves of seedlings [130]. 

The application of S-methylmethionine (SMM), a treatment to induce stress tolerance in plants, also stimulates the concentration of anthocyanins in maize seedlings and stems [131,132]. The SMM and salicylic acid (SA) pre-treatment preserved the photosynthetic activity under cold conditions, enhancing the anthocyanin content in the stalk and gene expression of the phenylpropanoid pathway members [131].

**Figure 7:**
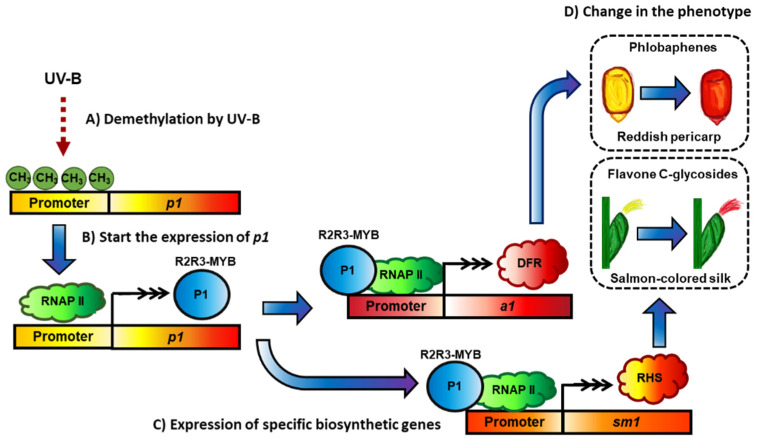
The locus *p1*, which regulates the biosynthesis of phlobaphenes and flavone C-glycosides, and its paramutation phenomenon. (**A**) UV-B produces the gene promoter demethylation of p1, with a consequent lower methylation level in the p1 promoter. (**B**) After demethylation, the p1 gene is expressed, and the P1 protein can function as a transcription factor. (**C**) P1 regulates a1 expression, leading to phlobaphene biosynthesis, and also activates essential genes for the flavone C-glucosides, such as sm1, which express the glucose 4,6-dehydratase (RHS). (**D**) The expression of these enzymes modifies the plant phenotype.

Another well-studied mechanism of regulation for the members of the MBW complex is the phenomenon of paramutation (Figure 7). Paramutation refers to the gene silencing that is heritable without changes in the DNA sequence between two alleles in the same locus [133]. The genes reported to present this phenomenon are *b1* [134], *r1* [102], *p1* [101], and *pl1* [100]. These genes have in common an upstream region, whose main characteristic is inverted repeat sequences that are transcribed into siRNA [133].

This production of siRNA depends on the *mop1* gene, which codes for an RNA-dependent RNA polymerase that is necessary to epigenetically silence a paramutable allele [103]. The mechanism for this silencing, produced by methylation of cytosine and chromatin modification, is still an actively studied topic [135]. External stimuli can also affect the epigenetic marks. UV-B can reduce the methylation of the promoter of *p1* of high-altitude landraces compared to inbred lines and induces higher expression of *p1* on leaves, enhancing the plant capacity to overcome the photo-oxidative stress related to high altitudes [3]. 

Other recently studied mechanisms of flavonoid regulation are those mediated by non-coding RNAs. Although this information is limited to maize, data obtained from another species may suggest a similar regulation mechanism. For example, the expression of long non-coding RNA in carrot (*Daucus carota*) or sea buckthorn (*Hippophae rhamnoides*) indicates that the MYB mRNA is not degraded by miRNA [136,137]. Thus, the MYB protein could integrate into the MBW complex, which is necessary for anthocyanin accumulation. In *Arabidopsis thaliana*, the expression of non-coding RNAs favors anthocyanin expression under abiotic stresses, such as phosphate or nitrogen deficiency [138]. Therefore, we could hypothesize that non-coding RNAs play a role in maize by regulating the MYB homologs, c1 and b1, of the MBW complex under an environmental stimulus. 

## 4. Cyanidin-3-*O*-Glucoside, One of the Most Abundant Flavonoids in Maize, and Its Effects on Human Health 

In recent years, the efforts of research have been focused not only on understanding the role of flavonoids in plant physiology and the flavonoid biosynthetic pathways, but also on the identification and elucidation of the action mechanisms linked to the potential health benefits attributed to these natural compounds. Noteworthily, flavonoids are present in regularly consumed plant foods such as pigmented maize and berry fruits and therefore represent an interesting therapeutic approach, collaterally covering nutritional aspects and inducing beneficial biological effects. 

Anthocyanins represent a broad-spectrum group of flavonoid compounds present in nature (>700), and they are the most abundant flavonoids present in pigmented maize varieties. Previously, we have reviewed and discussed the myriad of positive effects attributed to anthocyanin-enriched extracts, which include antioxidative, antimicrobial, antifungal, antihyperglycemic, antitumoral, and anti-inflammatory activities [139]. Cyanidin 3-*O*-glucoside is the most extensively studied anthocyanin. This flavonoid compound is usually considered one of the most abundant in edible plants exhibiting deep-purple coloration. In Table 5, we provide an update on published data supporting the beneficial health effects as well as molecular targets and pathways influenced by this plant-derived compound.

## 5. Conclusions

The flavonoid profile corresponds to a specific allele combination that dictates which compounds will be present in the tissue. However, the environmental factors are the ones that deliver signals, through phytohormones, regulating their concentration. Those factors have an impact not only on the survival of the plant but also on food science in terms of nutritional value and health benefits. 

Considering the importance of maize anthocyanin genetics in producing a better pigmented maize variety, maize should be selected according to the intended food processes. For example, selecting deep-colored grains with pigmentation in the pericarp (and the functional alleles) is not adequate when the food product requires a nixtamalization process to obtain maize dough. In fact, seeds for red or purple maize dough differ only in the gene *pr1*. 

Recessive alleles or new mutations in genes controlling the upregulation of the enzymatic machinery or initial enzymatic steps of a flavonoid pathway produce colorless tissue. Thus, this allows the inclusion of other chemical pigments, such as carotenoids or chlorophylls, to be responsible for the tissue color instead of flavonoids. In addition, maize has a natural capacity to produce grain enriched with anthocyanins, but consumers normally prefer to consume maize without anthocyanins. For centuries, white or yellow seeds have seemed to be more appealing to human populations worldwide, except in some places that grow and conserve the pigmented maize landraces. Previous health research has demonstrated the effectiveness of anthocyanin consumption in preventing or managing diseases related to oxidative stress, although more studies are needed to confirm some proposed action mechanisms of flavonoids in human health.

In this review, we have provided updated information regarding colorful phytochemicals, including anthocyanins, which exert first a physiological role in the plant but may also trigger diverse biological responses with the potential to improve human health.

## Figures and Tables

**Table 1:** Summary of genes involved in the early steps of the maize flavonoid pathway.

Gene Name	Locus	Enzyme/Protein Name	EC	Reference
(*ZmPAL*)	m*	Phenylalanine ammonium lyase	4.3.1.24	[16]
(*ZmC4H*)	8L	Cinnamic acid 4-hydroxylase	1.14.14.91	[16,17]
*bm5* (*Zm4CL*)	5	4-Coumarate CoA ligase	6.2.1.12	[22]
*c2* (*ZmCHS*)	4L	Chalcone synthase	2.3.1.74	[24]
*whp1* (*ZmCHS*)	2L	Chalcone synthase	2.3.1.74	[25]
*chi1* (*ZmCHI*)	1L	Chalcone isomerase	5.5.1.6	[26]
*fht1* (*ZmF3H*)	2S	Flavonoid 3-dioxygenase	1.14.11.9	[27]
*pr1* (*ZmF3*′*H*)	5L	Flavonoid 3′-monooxygenase	1.14.14.82	[28]

EC code and locus were obtained from BRENDA [29] and MaizeGDB [30], respectively. The m* means multiple loci.

**Table 2:** Summary of anthocyanin genes in the maize flavonoid pathway.

Gene Name	Locus	Enzyme/Protein Name	EC	Reference
*pr1* (*ZmF3*′*H*)	5L	Flavonoid 3′-monooxygenase	1.14.14.82	[28]
*a1* (*ZmDFR*)	3L	Dihydroflavonol 4-reductase	1.1.1.219	[44]
-(*ZmLAR*)	-	Leucoanthocyanidin reductase	1.17.1.3	- *
*a2* (*ZmANS*)	5S	Anthocyanidin synthase	1.14.20.4	[50]
*bz1* (*ZmAGT*)	9S	Anthocyanidin 3-*O*-glucosyltransferase	2.4.1.115	[51]
*aat1* (*Zm3MAT*)	1L	Malonyl-CoA: anthocyanin 3-*O*-glucoside-6′′-*O*-malonyltransferase	2.3.1.171	[52]
*omt1 and omt4*- (*ZmAOMT*)	4L	Anthocyanin S-adenosyl-l-methionine-dependent *O*- methyltransferase	2.1.1.267	[53,54]
*bz2* (*ZmGST*)	4L	Glutathione-S-transferase	2.5.1.18	[55]
*mrpa3* (*ZmABC3*) *mrpa4* (*ZmABC4*)	9S 1S	Multidrug resistance-associated protein or ATP-binding cassette transporter	7.6.2.2	[56]

* Not found yet in maize. EC code and locus were obtained from BRENDA [29] and MaizeGDB [30], respectively.

**Table 3:** Summary of flavonol and flavone C-glycoside genes in the maize flavonoid pathway.

Gene Name	Locus	Enzyme/Protein Name	EC	Reference
*fls1 (ZmFLS1*) *fls2 (ZmFLS2*)	5L 5L	Flavonol synthase	1.14.20.6	[1]
*fnsi1* (*ZmFNSI1*) *fnsi2* (*ZmFNSI2*)	1S 1S	Flavone synthase I	1.14.20.5	[84]
*fnsii1* (*ZmFNSII1*)	10L	Flavone synthase II	1.14.19.76	[2]
fns1 (*ZmF2H1*)	9L	Flavanone 2-hydroxylase	1.14.14.162	[85]
*cgt1* (*ZmCGT*)	6L	UDP-glucose:2-hydroxyflavanone C-glucosyltransferase	2.4.1.360	[86]
*sm2* (*UGT91L1*)	2L	flavonol-3-*O*-glucoside L-rhamnosyltransferase	2.4.1.159	[87]
*sm1* (*ZmRHS1*)	6L	Glucose-4,6 dehydratase	4.2.1.76	[43]

EC code and locus were obtained from BRENDA [29] and MaizeGDB [30], respectively.

**Table 4:** Genes that control the anthocyanin accumulation in maize.

Gene Name	Family	Locus	Function	Regulates	Expression of Functional Allele	Paramutation
*c1* (*ZmMYB1*)	R2R3-MYB	9S	+	*a1*, *a2*, *bz1*, *bz2*, and *c2*	Aleurone and scutellum	Not known
*pl1* (*ZmMYB2*)	R2R3-MYB	6L	+	Same as *c1*	Sheaths, pericarp, husk, culms, cob, and anther glumes	Yes
*p1* (*ZmMYB3*)	R2R3-MYB	1S	+ (works alone)	*a1* and *ch1*	Pericarp, silks, cob, and anther glumes	Yes
*p2* (*ZmMYB55*)	R2R3-MYB	1S	+ (works alone)	Same as *p1*	Silks and anther glumes	Not known
*r1* (*ZmbHLH1*)	bHLH	10L	+	*a1*, *a2*, *bz1*, *bz2*, and *c2*	Anthers, brace roots, leaf blade tips, aleurone. and scutellum	Yes
*b1* (*ZmbHLH2*)	bHLH	2S	+	Same as *r1*	Sheaths, pericarp, husk, culms, cob, and anther glumes	Yes
*in1*	bHLH	7S	-	*r1*	Competition against *r1*	Not known
*pac1* (*ZmWD40*)	WD40	5L	+	*a1*, *a2*, *bz1*, *bz2*, and *c2*	Any anthocyanin pigmented tissue	Not known
*a3*	-	3L	-	*b1*	Dominant inhibitor	Not known

Symbols: + = activation, − = inhibition. References: [30,96,99,100,101,102,103].

**Table 5:** Beneficial health effects and action mechanisms reported for cyanidin 3-*O*-glucoside (C3G).

Biological Effects	Type of Study	Dose, Time, and Model	Main Biological Findings	Ref.
Antitumoral	In vitro	10 and 20 µM; 24 h; human breast cancer MDA-MB-231 and Hs-578T cells	Attenuation of breast cancer-induced angiogenesis via inhibiting VEGF up-regulation of miR-124 reduces angiogenesis (inhibiting STAT3).	[140]
	In vitro	5, 10, 20, and 40 µM; 24 h; MDA-MB-231 and BT-549 cells	C3G induces reversion of EMT characterized by phenotype modulation with increased epithelial marker E-ca and ZO-1 and decreased mesenchymal marker vimentin, N-ca, and EMT-associated transcription factors Snail1 and Snail2.	[141]
	In vitro and in vivo	5, 20, 50, 150, 300, and 500 µM; 12, 24, 48, and 72 h; human breast cancer cells, melanoma cells, human embryonic kidney 293 cells, mouse and human primary melanocytes, and human samples of melanoma	C3G treatment arrested the cell cycle at the G2/M phase by targeting cyclin B1 (CCNB1) and promoted apoptosis via ERβ in both mouse and human melanoma cell lines.	[142]
	In vivo	10, 20, 40, and 80 µM; Chinese hamster ovary cells, human colon cancer cell lines, human breast cancer cell lines, and human melanoma cell line	C3G binds to talin (a key regulator of integrins and cell adhesion) and promotes the interaction of talin with β1A-integrin.	[143]
	In vitro	10 and 40 μM; 24 h; MDA-MB-231 and MDA-MB-468 breast cancer cells	The EMT inhibition is related to the upregulation of KLF4, which has been reported to be an EMT suppressor in breast cancer cells. The upregulation of KLF4 expression by C3G involves transcriptional suppression of FBXO32. It was found that FBXO32 acted as a promoter of EMT and cell migration/invasion.	[144]
	In vivo	Drosophila malignant RafGOFscrib −/− model. Purified C3G was added to standard food. Doses: C3G: 0.1 mg/mL or 0.4 mg/mL.	Purified C3G inhibited tumor growth invasion, distant migration and prolongs the survival of tumor flies. C3G inhibited tumor invasion by reducing the MMP1 activity and through JNK pathway.	[145]
	In vitro	HeLa cells by evaluating cell proliferation assay (C3G doses: 0–800 μg/mL) during 24, 48, and 72 h; apoptosis (Cy3G dose: 400 μg/mL), cell cycle, cell migration, and invasion evaluation (C3G dose: 400 μg/mL).	C3G combined with DPP induced apoptosis associated with the suppressed PI3K/AKT/mTOR signaling. DDP plus C3G treatment of HeLa cells can inhibit cell proliferation through cyclin D1 downregulation. Finally, this combined treatment could inhibit the migration and invasion associated with decreasing the protein of TIMP-1.	[146]
	In vivo	Induction of hepatic precancerous lesion (PCL) with diethylnitrosamine/2-acetylaminofluorene (DEN/2-AAF) in a Wistar rat model. C3G (not described source) at 10, 15, and 20 mg/kg/day. The measured parameters were alpha fetoprotein (AFP) levels and liver function biochemical analytes, and RNA panel differential expression was evaluated via qPCR. Histopathological examination of liver sections stained with H&E was also conducted.	AFP levels were significantly decreased in the three C3G doses. Decreased ALT levels and increased serum albumin were found after C3G treatment. Moreover, C3G treatment decreases, in a dose-dependent manner, the mRNA expression of long non-coding RNA MALAT1 and tubulin gamma 1 and increases the miR-125b levels. Histologically, less discriminated dysplastic nodules were exhibited by liver sections of rats treated with C3G.	[147]
	In vitro	C3G or its metabolite protocatechuic acid (PCA) were tested at the following doses: 100, 200, and 400 μM in HepG2 cells. Cell viability after C3G and PCA treatments, LDH release, and apoptosis in HepG2 cells in which cytotoxicity was induced using 2-amino-3-methylimidazo [4,5-f]quinoline (IQ) were evaluated using CCK-8, LDH release, and flow cytometry assays, respectively. Tandem mass tag (TMT)-based proteomics was utilized to characterize the proteins and pathways associated with the improvement after C3G and PCA treatment.	Exposure to IQ increased cytotoxicity and apoptosis in HepG2 cells, which were alleviated by C3G and PCA. C3G was more effective than PCA in protecting HepG2 cells against IQ-induced cytotoxicity and regulating the related signaling pathways. Proteomics and bioinformatics analyses and Western blot validation revealed that apoptosis-related signaling pathways played pivotal roles in the protective effect of C3G against the cytotoxicity of IQ. Moreover, XIAP was identified as a key target. XIAP acts as a potent apoptotic inhibitor by hampering the activation of caspases 3, 7, and 9. Molecular docking provided evidence that C3G affected the bindings of IQ and its carcinogenic metabolites to XIAP-BIR3 and contributed to the inhibition of apoptosis.	[148]
	In vitro	Cisplatin (DDP) dose: 5 μg/mL. C3G (>98% purity) dose: 400 μg/mL. Cervical cancer HeLa cells. Measurements of oxidative stress (CAT, SOD, and GSH-Px) and quantitation of gene expression of bax, bcl-2, Nrf2, and *Keap1* genes were performed.	C3G-DDP inhibited the activity of the antioxidant defense enzymes SOD, CAT, and GSH-Px. In parallel, C3G-DDP reduced GSH concentration while increasing the concentration of ROS and MDA. C3G-DDP reduces the expression of Nrf2 and Nrf2 target proteins: HO-1 and NQO1. Finally, C3G-DDP increased the mRNA expression ratio of bax/bcl-2 and activated the intrinsic apoptotic pathway of HeLa cells.	[149]
Antidiabetic and protection against complications of diabetes	In vitro and in vivo	1 and 5 µM; 4 h pre-treatment of ARPE-19 cells exposed to 30 µM 4-hydroxynonenal for 24 h. 50 (mg/kg)/day for 3 weeks (2 pre-illumination and 1 post-illumination) in rabbits in which retinal damage was induced by light exposure	Decreased apoptosis, lower senescence-associated beta-galactosidase, and lower VEGF release. Increased thickness of the neurosensory retina in rabbits exposed to light.	[150]
	In vitro	10 µM; 6 h; mouse colonic epithelial MCE301 cells	Higher gene expression of the Mg^2+^ transport carriers Trpm6 and Cnnm4.	[151]
	In vivo	10 and 20 (mg/kg)/day for 8 weeks in Sprague Dawley rats in which diabetes was induced with a 45 mg/kg streptozotocin dose.	Reduced fasting glycemia and insulin levels, decreased serum creatinine and BUN, and lower urinary albumin. Improved antioxidant enzyme and reduced cytokine levels. Decreased fibrosis and glomerulosclerosis in renal tissue.	[152]
	In vitro	100 µM; 24 h; human corneal epithelial cells (HCEC 6510) previously exposed to 10 µg/mL of LPS for 24 h	Reduced apoptosis and decreased production of cytokines.	[153]
	In vivo	1.6 mg/mL in drinking water (∼6.4 mg/day), for 3 or 20 weeks, in C57BL/6J male mice fed a low- or high-fat diet	Decreased weight gain for high-fat diet, improved glucose tolerance, reduced hepatic and plasma triglycerides, and modulated hepatic FGF21 levels.	[154]
	In vitro and in vivo	20 µM; 24 h; HUVEC cells exposed for 1 h to 100 ng/mL TNF-alpha before treatment with C3G. 50 or 100 mg/kg; 8 weeks; male New Zealand rabbits fed 8 weeks with a high-fat diet after balloon catheter injury was performed.	Reduced damage in the intima media; decreased levels of circulating cholesterol, low-density lipoprotein, and triglycerides; and increased high-density lipoprotein. Reduced levels of cytokines and lowered apoptosis rates. Higher expression of SIRT1.	[155]
	In vitro	20 µM; 48 h (+1 h pre-treatment); lens epithelial SRA01/04 cells exposed to 100 mM glucose and Sprague Dawley rat lens tissue exposed to 50 mM glucose.	Reduced apoptosis rates, decreased NFkB levels, and lowered Cox-2 protein expression. Decreased opacity of rat’s lens tissue.	[156]
	In vitro	5 and 10 µM pre-treatment; 24 h; 3T3-L1 cells and human SGBS cells exposed to 1 mM or 500 µM palmitate for 24 h	Reduced lipid content, lower PPARgamma and nuclear NFkB protein levels, improved levels of insulin signaling targets, and higher Adipoq gene expression.	[157]
	In vivo	10 and 50 µM; 24 h; HepG2 pre-treated with 400 µM palmitic acid and 400 µM oleic acid for 24 h. 50 mg/day; 8 weeks; male C57BL 6J mice previously fed an HFD for 4 weeks and 8 additional weeks of HFD during C3G treatment.	Reduced plasma and liver triglycerides, reduced fatty acid synthesis, lower fasting plasma glucose and insulin, higher cell glucose uptake, activation of PPAR-alpha.	[158]
Liver disease and hepato-protection	In vitro and in vivo	100 µM; 12 h; HepG2 or AML-12 cells co-treated with 400 µM palmitic acid 0.2% (*v*/*v*) of C3G in the HFD, 4 weeks (after 12 weeks of HFD), male mice fed an HFD for 16 weeks	Reduced liver steatosis, lower fasting glucose and insulin levels, reduced NLRP3 inflammasome, higher antioxidative enzyme levels, lower ROS levels, increased mitophagy.	[159]
	In vitro	5 µg/mL; 12 h; HepG2 cells pre-exposed to 4 µM hydrogen peroxide for 6 h	Decreased ROS levels, increased glutathione content, and higher catalase activity. Increased Nrf2 and Keap1 protein levels.	[160]
	In vitro	2.5–10 µM; 24 h; HepG2 cells previously treated with 400 µM hydrogen peroxide	Increased cell viability and antioxidative machinery. Decreased ROS, apoptosis rates, and apoptosis-related proteins.	[161]
	In vitro and in vivo	200 (mg/kg)/day; 8 weeks; male C57BL 6J mice fed an HFC diet and 5% ethanol drinking solution during the C3G treatment. HepG2 and FL83B cells were treated with SIRT1 inhibitor EX527 at 10 µM, for 4 h, and 1 µM C3G for an additional 20 h.	Reduced liver lipid content, lower levels of proinflammatory cytokines and inflammasome proteins, reduced NFkB protein expression and acetylation, and increased SIRT1 protein levels.	[162]
Colitis and gastrointestinal alterations	In vivo and in vitro	1 ug i.p. on days 0, 3, and 6 of model induction; C57BL 6J mice in which colitis was induced with drinking water containing 3.5% of dextran sulfate sodium for 7 days. 1 µg/mL; 24 h; peritoneal macrophages activated with 1 ug mL^−1^ of LPS.	Reduced cytokine gene expression in the colon, induction of Treg cells, and reduction of peritoneal CD169+ macrophages.	[163]
	In vivo	500 and 1000 mg/kg of diet; 8 weeks; male Wistar rats in which dysbiosis and intestinal damage were parallelly induced with 20 mg/kg 3-chloro-1,2-propanediol for 8 weeks	Improved histological features, modulation of gut microbiota.	[164]
	In vivo and in vitro	50, 100, or 200 µmol/kg; 3 days; female BALB c mice in which colitis was induced with 2.5 mg of 2,4,6-trinitrobenzen-osulfonic acid 12 h after the first dose of C3G. 50 and 100 µmol/L; 24 h pre-treatment; LPS-induced Caco-2 cells with 100 ng/mL for 24 h.	Prevention of histological damage, reduction of proinflammatory cytokines, and suppression of nitric oxide production.	[165]
	In vitro	10 or 20 µmol/L; 24 h pre-treatment; Caco-2 cells induced with palmitic acid 100 µmol/L for 6 h	Decreased nuclear NFkB, reduction of cytokine IL6 and IL8 gene expression and COX2 protein, decrease in ROS, and increase in Nrf2 levels.	[166]
Neuroprotective	In vitro	2.5, 5, or 10 µmol/L; 4 h pre-treatment; microglial BV2 (macrophage) cells stimulated with 1 μg/mL LPS for 24–48 h	Decreased cytokine levels, reduced iNOS mRNA levels and lower NO production, suppression of NFkB activation and p38 signaling pathway, decreased neurotoxicity and apoptosis in PC12 cells exposed to conditioned media from LPS-activated BV2 cells.	[167]
	In vitro	0.05, 0.1, 0.25, 0.5, or 1 µmol/L; 24 h pre-treatment; HT22 neuronal cells exposed to 5 mM glutamate for 18 h	Reduction of apoptosis, decrease in ROS, increase in Nrf2 levels and antioxidative gene expression, reduction of ER stress biomarkers.	[168]
	In vitro	1, 3, or 9 µmol/L; 24–48 h; PC12 neuronal cells exposed in parallel to amyloid beta fibrils	Increased cell viability, decreased necrosis, reduced ROS levels.	[169]
	In vitro	30 mg kg-1 day-1; 38 weeks; APPswe/PS1dE9 mice modeling Alzheimer’s disease	Differential gene expression in the spleen of the treated animals, including upregulation of antioxidant and immune system-related molecular targets.	[170]
Reproductive health	In vitro	5, 20, 40, 80, or 160 µmg/L; 2 h pre-treatment; Leydig R2C cells exposed to 44.8 µmol/L cadmium sulfate for 24 h	Increased cell viability, reduced ROS levels, protection of mitochondrial potential, increased StAR protein and progesterone levels.	[171]
	In vivo	500 mg/kg of chow diet; 10, 20, or 30 days; Kunming male mice treated with 5 (mg/kg)/day of cadmium chloride	Decreased levels of circulating FSH and testosterone, increased LH circulating levels, differential modulation of gene expression in the hypothalamus, increased expression of proteins involved in testosterone biosynthesis.	[172]
Respiratory system, antiviral, and anti-SARS-CoV2	In vivo	Diet containing 0.4% C3G (~1.2 mg/day); 25 days; asthma model of BALB/c mice sensitized to ovalbumin intraperitoneally (20 μg on days 0, 7, and 14) and nasally (1% aerosols on days 21–25)	Decreased number of peripheral eosinophils; reduced inflammatory infiltration in the lungs; lower levels of IL-4, IL-5, and IL-13; inhibition of IL-4Ra-STAT6 pathway.	[173]
	In silico and in vitro	3–200 µmol/L; papain-like protease assay for determination of deubiquitinase activity	The molecular docking prediction showed a potential binding activity to the papain-like protease of SARS-CoV-2, concentration-dependent inhibition of papain-like protease deubiquitinase activity.	[174]
	In silico and in vitro	3–200 µmol/L; papain-like protease assay for determination of total protease activity	Molecular docking prediction of binding to the papain-like protease of SARS-CoV2. Concentration-dependent inhibition of papain-like protease total protease activity.	[175]
	In vivo and in vitro	200 or 400 mg/kg bw; oral administration from days 2–28; Sprague Dawley male rats injected intraperitoneally with monocrotaline 60 mg/kg bw on day 1 to induce a model of pulmonary artery hypertension. 10 or 20 µmol/L; 24 h pre-treatment; cells induced with TGF-beta1 8 ng/mL for additional 24 h.	Reduction of hemodynamic indicators of pulmonary artery hypertension, improved histological features and blood oxygenation, reduction of cytokines levels, reduced markers of proliferation in PASMC, inhibition of TGF-beta1-p38 MAPK-CREB signaling pathway.	[176]
Anti-inflammatory and immune system modulation	In vivo and in vitro	25 mg/kg; two tail-vein injections per week for a total of six injections starting ten days after the secondary immunization; Sprague-Dawley male rats in which arthritis was induced by three injections of bovine type II collagen. 25, 50, or 100 µmol/L; 24–48 h; rheumatoid arthritis synovial fibroblasts and mononuclear cells obtained from patients.	Increased Treg cells and decreased CD38+ NK cell proportion in blood and synovial fluid in murine model, increased apoptosis and decreased proliferation in human rheumatoid arthritis synovial fibroblasts, decrease in proinflammatory cytokines.	[177]
	In vivo	10 (mg/kg)/day; 15 weeks; spontaneously hypertensive male rats and Wistar-Kyoto rats.	No differences were observed either in the spleen weights or in the proportions of splenic T-cells and helper T-cells; modulation of CD62Lhi, CD62Llo, CD62L-, CD25+, and T-reg cells dependent on the genotype.	[178]
	In vitro and in vivo	25, 50, 100, and 250 µmol/L, RBL-2H3 cells sensitized with anti-DNP IgE and exposed to DNP-BSA antigen 100 and 200 µmol/kg bw, orally administered 1 h before antigen exposure, and 40 mg/kg bw, intravenous administration 1 h before antigen challenge, male ICR mice sensitized with anti-DNP IgE (100 ng injection in the ear) 24 h before the experiment and then challenged with DNP-BSA antigen (140 µg/mouse).	Dose-dependent inhibition of histamine and beta-hexosaminidase release, decreased ear tissue response (measured as extravasation) after antigen challenge.	[179]
Other studies	In vitro	80 µmol/L; 2 h pre-treatment; primary human dermal fibroblast irradiated with 12 J/cm^2^ UVA light and treated with 3-methyladenine	Decreased apoptosis, increased expression of autophagy markers, reduced ROS levels.	[180]
	In vivo	100 mg/kg body weight, oral administration before induction; Wistar rats injected with 1 mL/kg of 5% taurocholate to induce a model of severe acute pancreatitis	Increased colonic motility, decreased serum levels of H_2_S and pro-inflammatory cytokines, activation of mTOR signaling, reduced protein levels of cystathionine-gamma-lyase.	[181]
	In silico	Molecular modeling to assess for potential interactions between C3G and the advanced glycation end product receptor and its ligands	The results suggest a potential interaction and subsequent inhibition of the receptor for advanced glycation end products.	[182]
	In vitro	25–400 µM; 24–72 h; primary human osteoblasts and MC3T3-E1 osteoblast murine cell line	Increased cell proliferation, increased mineralization activity, activation of ERK1/2 signaling pathway, increased osteocalcin protein and mRNA levels.	[183]
	In vitro	1.25, 2.5, and 5 µmol/L; 24 h co-treatment or 2 h pre-treatment; primary human articular chondrocytes exposed to advanced glycation end products 10 µg/mL for 24 h (parallel to C3G treatment) or 10 min (after 2 h pre-treatment).	Reduced protein and mRNA expression levels of matrix metalloproteinases, decreased NF-kB signaling, reduced ERK/MAPK signaling activation.	[184]
	In vitro	20 µmol/L; six-day treatment renewed every 48 h; human amniotic epithelial cells	Differential modulation of genes including targets involved in adipocyte differentiation and muscle activity.	[185]

EMT = epithelial–mesenchymal transition, CCNB1 = cyclin B1, DDP = cis-diamminedichloroplatinum, CAT = catalase, SOD = superoxide dismutase, GSH-Px = glutathione peroxidase, Nrf2 = nuclear factor erythroid 2-related factor-2, Keap1 = Kelch-like ECH-associated protein 1, HO-1 = heme oxygenase-1, NOQ1 = NAD(P)H quinone dehydrogenase 1, PCA = protocatechuic acid, MDA = malondialdehyde, TMT = tandem mass tag, VEGF = vascular endothelial growth factor.

## Data Availability

Not applicable.
